# Engineering of xylose metabolic pathways in *Rhodotorula toruloides* for sustainable biomanufacturing

**DOI:** 10.1093/femsyr/foaf029

**Published:** 2025-06-11

**Authors:** Hyunjoon Oh, Hyun Gi Koh, Suk-Chae Jung, Quanhui Ye, Sujit Sadashiv Jagtap, Christopher V Rao, Yong-Su Jin

**Affiliations:** Department of Food Science and Human Nutrition, University of Illinois at Urbana-Champaign, Urbana, IL 61801, United States; DOE Center for Advanced Bioenergy and Bioproducts Innovation, University of Illinois at Urbana-Champaign, Urbana, IL 61801, United States; Department of Biological and Chemical Engineering, Hongik University, Sejong 30016, Republic of Korea; Department of Bioengineering, University of Illinois Urbana-Champaign, Urbana, IL 61801, United States; DOE Center for Advanced Bioenergy and Bioproducts Innovation, University of Illinois at Urbana-Champaign, Urbana, IL 61801, United States; Carl R. Woese Institute for Genomic Biology, University of Illinois at Urbana-Champaign, Urbana, IL 61801, United States; DOE Center for Advanced Bioenergy and Bioproducts Innovation, University of Illinois at Urbana-Champaign, Urbana, IL 61801, United States; Department of Chemical and Biomolecular Engineering, University of Illinois Urbana-Champaign, Urbana, IL 61801, United States; DOE Center for Advanced Bioenergy and Bioproducts Innovation, University of Illinois at Urbana-Champaign, Urbana, IL 61801, United States; Carl R. Woese Institute for Genomic Biology, University of Illinois at Urbana-Champaign, Urbana, IL 61801, United States; Department of Chemical and Biomolecular Engineering, University of Illinois Urbana-Champaign, Urbana, IL 61801, United States; Department of Food Science and Human Nutrition, University of Illinois at Urbana-Champaign, Urbana, IL 61801, United States; DOE Center for Advanced Bioenergy and Bioproducts Innovation, University of Illinois at Urbana-Champaign, Urbana, IL 61801, United States; Carl R. Woese Institute for Genomic Biology, University of Illinois at Urbana-Champaign, Urbana, IL 61801, United States

**Keywords:** *Rhodotorula toruloides*, metabolic engineering, biomanufacturing, lignocellulosic hydrolysate, xylose metabolism, D-arabitol

## Abstract

The oleaginous yeast *Rhodotorula toruloides* is a promising microbial cell factory for the sustainable production of biofuels and value-added chemicals from renewable carbon sources. Unlike the conventional yeast *Saccharomyces cerevisiae, R. toruloides* can naturally metabolize xylose, the second most abundant sugar in lignocellulosic hydrolysates. However, its native xylose metabolism is inefficient, characterized by slow xylose uptake and accumulation of D-arabitol. Moreover, despite its phenotype, research on the enzymes involved in xylose metabolism has yet to reach a consensus. Therefore, this review provides a comprehensive analysis of the non-canonical xylose metabolism in *R. toruloides*, focusing on the properties of key enzymes involved in xylose metabolism. Native xylose reductase and xylitol dehydrogenase exhibit broad substrate promiscuity compared to their counterparts in the xylose-fermenting *Scheffersomyces stipitis*. Additionally, the absence of xylulokinase expression under xylose-utilizing conditions redirects metabolism toward D-arabitol accumulation. Consequently, D-arabitol dehydrogenases and ribulokinase play essential roles in the xylose metabolism of *R. toruloides*. These findings highlight the fundamental differences between *R. toruloides* xylose metabolism and the oxidoreductase pathways observed in other xylose-fermenting yeast, providing insights for metabolic engineering strategies to improve xylose utilization and enhance bioconversion of cellulosic hydrolysates to different bioproducts by *R. toruloides*.

## Teaser

This review examines the unique xylose metabolism of *Rhodotorula toruloides* and explores strategies to improve its efficiency for sustainable biomanufacturing.

## Introduction

With recent advances in biotechnology, yeast fermentation has expanded beyond traditional food applications to include the production of valuable biofuels and chemicals through engineered biosynthetic pathways, contributing to a greener environment and a more sustainable bioeconomy (Nielsen et al. 2022, Yang et al. 2022). While the well-established baker’s yeast *Saccharomyces cerevisiae* has been extensively used for such applications, non-conventional yeasts such as *Rhodotorula toruloides* are increasingly gaining attention for their metabolic versatility and potential in industrial bioproduction (Park et al. 2018). Despite these developments, current commercial biomanufacturing still largely depends on feedstocks derived from food sources such as cornstarch and sugarcane. Therefore, reducing dependence on food-based raw materials offers a greener and more sustainable alternative. Lignocellulosic biomass, sourced from non-edible crop residues and whole non-edible plants, serves as a viable feedstock for the sustainable production of a wide range of bioproducts (Rooni et al. 2017).

Yeast fermentation with lignocellulosic biomass faces several challenges. Lignocellulosic biomass is resistant to microbial and enzymatic degradation, requiring harsh pretreatment and enzymatic hydrolysis to release fermentable carbon sources such as glucose, xylose, and acetic acid (Zoghlami and Paes 2019). Xylose is the second most abundant sugar in lignocellulosic hydrolysates (Koh et al. 2024). Since feedstock contributes 50%–70% of the total production costs in lignocellulosic bioconversion, the most economically viable process should utilize all available sugars, not just glucose (Gong et al. 2022). However, *Sa. cerevisiae*, the most commercially used yeast, cannot utilize xylose without metabolic engineering (Lee et al. 2021). As a result, *Sa. cerevisiae* leaves xylose unutilized in the medium, limiting the full production potential of lignocellulosic hydrolysates (Kim et al. 2013).

**Figure 1.**
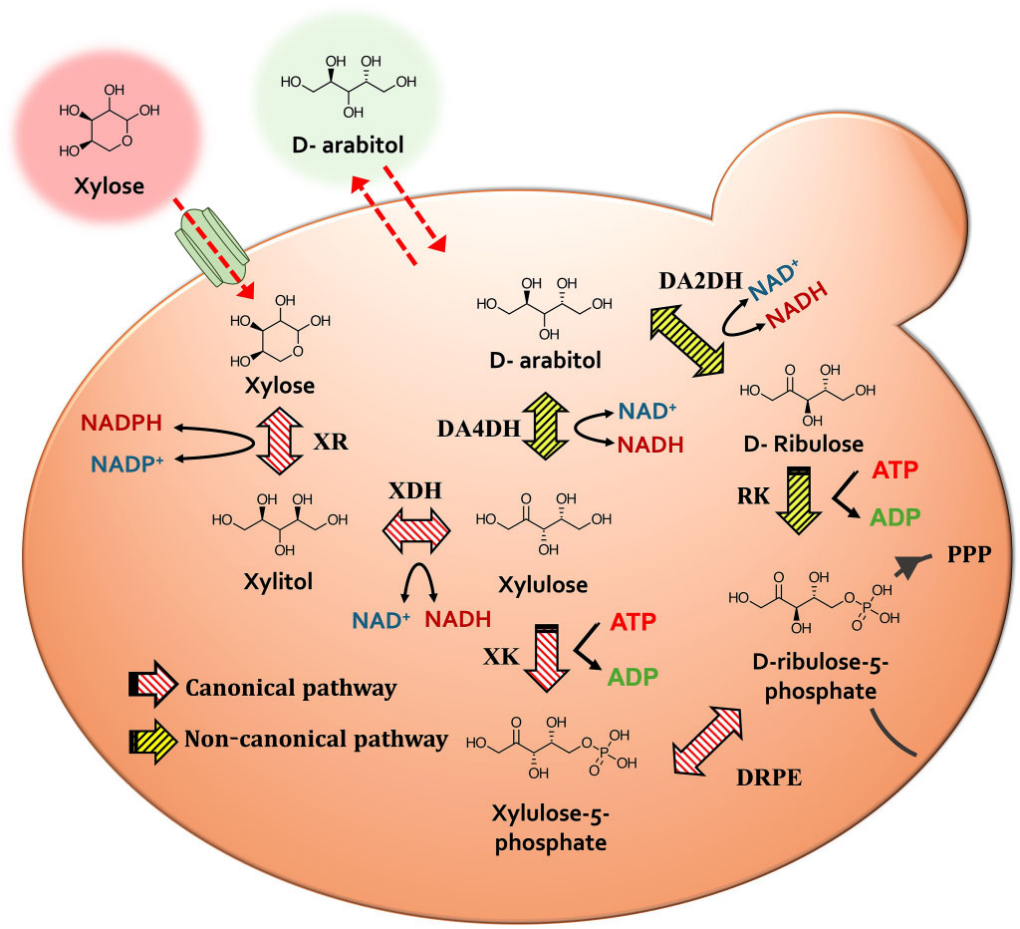
A schematic overview of the endogenous xylose assimilation pathway of *R. toruloides*. XR: xylose reductase, XDH: xylitol dehydrogenase, XK: xylulokinase, DA4DH: D-arabitol-4-dehydrogenase, DA2DH: D-arabitol-2-dehydrogenase, RK: ribulokinase, DRPE: D-ribulose-5-phosphate 3-epimerase, PPP: pentose phosphate pathway.

Most xylose-fermenting yeasts metabolize xylose via the oxidoreductase pathway under aerobic conditions. This canonical pathway consists of two enzymes, xylose reductase (XR) and xylitol dehydrogenase (XDH), converting xylose into xylulose via xylitol as an intermediate (Fig. 1). Xylulokinase (XK) then phosphorylates xylulose into xylulose-5-phosphate, which enters central carbon metabolism through the pentose phosphate pathway (PPP; Kwak and Jin 2017). *Scheffersomyces* (formerly *Pichia*) *stipitis* is a well-known xylose-fermenting yeast with an oxidoreductase pathway (Jeffries et al. 2007). Introducing the three genes coding for XR, XDH, and XK from *Sc. stipitis* to non-xylose-fermenting yeasts such as *Sa. cerevisiae* and *Yarrowia lipolytica* has enabled xylose utilization (Kim et al. 2013, Ledesma-Amaro et al. 2016, Yook et al. 2025).

*Rhodotorula toruloides* can utilize xylose without any genetic modifications (Wiebe et al. 2012, Xu and Liu 2017). In addition to xylose, this yeast can metabolize a wide range of substrates, including glycerol, organic acids, lignin-derived aromatics, and fatty acids, and grow to high cell densities, making it a promising candidate for lignocellulosic bioconversion (Park et al. 2018, Spagnuolo et al. 2019). *Rhodotorula toruloides* can accumulate lipids more than 70% of its dry cell weight and has been engineered to produce a wide range of chemicals, such as modified lipids, acetyl–CoA-derived products, terpenes, and organic acids (Zhang et al. 2016, Yaegashi et al. 2017, Cao et al. 2022, Schultz et al. 2022, Liu et al. 2023). Furthermore, it is a natural producer of commercially valuable carotenoids, including torularhodin, torulene, and β-carotene (Malisorn and Suntornsuk 2008, Lee et al. 2014). These traits have fueled extensive research on *R. toruloides* over the past decade (Wen et al. 2020).

Recent advances in genetic engineering tools for *R. toruloides* can be applied to enhance xylose metabolism further. Genes of interest can be expressed with strong and constitutive promoters native to *R. toruloides* (Nora et al. 2019). A Golden Gate assembly toolkit named *R. toruloides* Efficient Zipper has been developed, enabling modular cloning of multiple genes for integration into *R. toruloides* (Koh et al. 2025). Cas9 systems for precise genome editing have also been developed for *R. toruloides*, showcasing efficient knockouts of multiple genes (Jiao et al. 2019, Otoupal et al. 2019, Schultz et al. 2019). However, targeted knock-in integration is challenging, as *R. toruloides* predominantly fixes the double-strand breaks from Cas9 using non-homologous end joining (NHEJ), rather than homology-directed repair (HDR; Koh et al. 2014, Lyu et al. 2023). To enhance the HDR efficiency of NHEJ-dominant yeasts, a new system named Lowered Indel Nuclease system Enabling Accurate Repair was developed, using a linear DNA fragment containing a Cas9-sgRNA cassette and a donor DNA (Ploessl et al. 2022). Moreover, an *in silico* platform for finding integration sites on the genome named COmputational Pipeline for the Identification of CRISPR/Cas-facilitated intEgration Sites (CRISPR-COPIES) has been developed (Boob et al. 2024). Using these tools, 12 stable integration sites in *R. toruloides* were identified and tested, enabling efficient and stable multiplex-targeted gene integrations (Xu et al. 2025). Given these attributes, *R. toruloides* holds significant potential for producing biofuels and high-value chemicals from lignocellulosic biomass.

Although *R. toruloides* can naturally assimilate xylose, its xylose metabolism is less efficient than its glucose metabolism due to the accumulation of D-arabitol from xylose as a byproduct (Jagtap and Rao 2018, Tiukova et al. 2019). Reported glucose consumption rates range from 0.42 to 0.60 g l^−1^ h^−1^, whereas xylose consumption rates are lower, reported at 0.29 and 0.35 g l^−1^ h^−1^ (Wiebe et al. 2012, Tiukova et al. 2019, Jagtap et al. 2021). A major inefficiency in xylose metabolism is the accumulation of D-arabitol, which can reach 23 g l^−1^ from 70 g l^−1^ xylose and up to 33 g l^−1^ from 150 g l^−1^ xylose (Jagtap and Rao 2018, Pinheiro et al. 2020). Unlike the canonical oxidoreductase pathway for xylose metabolism in yeast, which does not produce D-arabitol, *R. toruloides* appears to employ an alternative metabolic route, a deviation from the canonical xylose utilizing pathway (Fig. 1). Indeed, recent findings suggest that D-arabitol dehydrogenases and ribulokinase (RK) play essential roles in its xylose metabolism (Jagtap et al. 2021, Adamczyk et al. 2023).

This review explores the non-canonical xylose metabolism of *R. toruloides* by examining key enzymes involved in xylose utilization. It compiles the genes associated with xylose metabolism, with a focus on deviations from the canonical oxidoreductase pathway and the properties of the enzymes making the deviations. Beyond providing a comprehensive overview of xylose metabolism in *R. toruloides*, this review also discusses metabolic engineering strategies to enhance xylose metabolism for producing high-value chemicals from lignocellulosic hydrolysates.

### Xylose transporter

The first step in xylose metabolism is transporting it across the cell membrane into the cytoplasm using specific transporters. While xylose transporters in *R. toruloides* are not well characterized, several genes annotated as sugar transporters are upregulated under xylose-utilizing conditions. In *R. toruloides* CBS14, eight transporters exhibited elevated protein levels under xylose conditions compared to glucose. Among them, Rhto_01630, annotated as a major facilitator superfamily (MFS) monosaccharide transporter, showed the highest upregulation with a 338-fold increase. Its National Center for Biotechnology Information (NCBI) accession number is XP_016272689 (Tiukova et al. 2019). Similarly, in *R. toruloides* IFO0880, multiple genes annotated as sugar transporters showed significant transcriptional increases: RTO4_10452 (annotated as general substrate transporter; NCBI accession number PRQ70912) with a 37-fold increase, RTO4_10804 (annotated as hexose transport-related protein; NCBI accession number PRQ70647) with a 138-fold increase, RTO4_12389 (annotated as MFS domain-containing protein; NCBI accession number PRQ76971) with a 106-fold increase, and RTO4_12976 (annotated as general substrate transporter; NCBI accession number PRQ75954) also with a 106-fold increase. Furthermore, the transcription levels of all the upregulated transporters decreased under glucose conditions, with RTO4_10804 (annotated as hexose transport-related protein) exhibiting the most significant reduction by 243-fold (Coradetti et al. 2018, Jagtap et al. 2021). These observations suggest that some of these upregulated sugar transporters might function as xylose transporters, although gene deletion experiments will be necessary to confirm their roles in xylose uptake.

However, those sugar transporters may exhibit low affinities for xylose. For example, RTO4_10452 (annotated as general substrate transporter) has a high similarity with the glucose transporter Hgt1 from *Kluyveromyces lactis*. As Hgt1 is a high-affinity glucose transporter showing 30% amino acid identity with the hexose transporter (HXT) family of *S. cerevisiae*, its affinity for xylose might not be high (Billard et al. 1996). RTO4_10804 (annotated as hexose transport-related protein) shows similarity with the sugar and polyol transporter *GsSPT1* from the algae *Galdieria sulphuraria*. Spt1 showed broad substrate specificities for glucose, fructose, ribose, and mannitol (Schilling and Oesterhelt 2007). RTO4_12389 (annotated as MFS domain-containing protein) is similar to fructose/glucose uniporter *ZrFfz2* from *Zygosaccharomyces rouxii* (Leandro et al. 2011). Lastly, RTO4_12976 (annotated as general substrate transporter) is similar to the glycerol proton symporter Stl1p from *Sa. cerevisiae* (Ferreira et al. 2005).

Collectively, these findings suggest that *R. toruloides* might harbor sugar transporters capable of transporting xylose. It remains uncertain whether *R. toruloides* has specific transporters dedicated to xylose.

Xylose transport in the presence of glucose is crucial in improving cellulosic sugar utilization, as lignocellulosic hydrolysates contain both glucose and xylose (Koh et al. 2024). However, in *S a. cerevisiae*, glucose represses xylose transport, so only when glucose is fully consumed, can yeast consume xylose (Ha et al. 2010). *Rhodotorula toruloides* IFO0880 also shows this sequential consumption of glucose and xylose (Coradetti et al. 2023). In *S a. cerevisiae*, two glucose transporters, Snf3 and Rgt2, are proposed to be glucose sensors (Ozcan 2002). Snf3/Rgt2 is involved in transcriptional regulation to fine-tune HXT expression levels based on hexose availability (Kayikci and Nielsen 2015). In *R. toruloides*, RTO4_11990 (annotated as putative glucose transporter; NCBI accession number PRQ76572) has a similar sequence with Snf3/Rgt2. RTO4_11990 is also related to a high-affinity glucose transporter, HGT-1, from *Neurospora crassa* (Coradetti et al. 2023). RTO4_11990 is predicted to be a transmembrane protein (Kim et al. 2020). However, the deletion mutant of RTO4_11990 did not allow simultaneous co-consumption of glucose and xylose. Surprisingly, the mutant outperformed the wild type on xylose as a sole carbon source. The mutant had faster xylose consumption, with about five-fold higher biomass formation (Coradetti et al. 2023). Further investigation on glucose repression mechanism in *R. toruloides* is critical in allowing simultaneous consumption of glucose and xylose.

The introduction of heterologous sugar transporters without glucose inhibition can be a promising metabolic engineering strategy to improve the utilization of cellulosic sugars by *R. toruloides*. For example, introducing a xylose transporter *Sp*Xut1 from *Spathaspora passalidarum* to *Sc. stipitis* achieved efficient co-utilization of glucose and xylose. By doing so, the authors were able to improve shikimate production, which requires substrates from both glycolysis and PPP from a mixed sugar fermentation (Gao et al. 2023). Similarly, the transporter LST1_205437 from an oleaginous yeast *Lipomyces starkeyi* partially alleviated glucose repression in *Sa. cerevisiae*, and a plant-derived transporter, *AtSWEET7* from *Arabidopsis thaliana*, also enabled simultaneous co-consumption of glucose and xylose (Kuanyshev et al. 2021).

### Xylose reductase

Once xylose is imported into the cytosol, xylose is reduced into xylitol by XR (Son et al. 2018). When compared with glucose conditions, intracellular xylitol level increased by 122-fold when *R. toruloides* is grown on xylose (Jagtap et al. 2021). This suggests that sufficient xylose reductase activities exist in xylose-utilizing *R. toruloides*.

*Rhodotorula toruloides* has multiple enzymes that might act as XR. RTO4_9774 (annotated XR; NCBI accession number PRQ71659) showed a 2.6-fold increase in mRNA levels on xylose conditions (Jagtap et al. 2021). It is a homolog of L-glyceraldehyde reductase in *Aspergillus niger*, showing a strong affinity toward D-galacturonic acid. It also shows a comparable Michaelis constant on xylose and is hypothesized to be a promiscuous pentose reductase (Protzko et al. 2019). The deletion mutant of RTO4_9774 grown on xylose as a sole carbon source exhibited slower growth and lower biomass formation compared to the wild type. However, the deletion of RTO4_9774 did not abolish the growth on xylose, which eludes the presence of multiple XRs in *R. toruloides* (Adamczyk et al. 2023). RTO4_11553 (NCBI accession number PRQ69900), identified to be an aldose reductase, has a specificity for both galactose and xylose. While RTO4_11553 was biochemically characterized as the key enzyme in galactose metabolism, it might have a promiscuous XR activity (Jagtap et al. 2019). A mild 1.2-fold increase in transcription levels on xylose supports its potential involvement in xylose metabolism (Jagtap et al. 2021). Based on the multi-omics study, RTO4_11882 (annotated to be a glycerol 2-dehydrogenase; NCBI accession number PRQ69861) and RTO4_13562 (annotated to be an alcohol dehydrogenase; NCBI accession number PRQ75505) have been proposed as potential pentose reductases (Kim et al. 2020). Putative enzymes proposed to have XR activities can be found in Table 1.

**Table 1.** *Rhodotorula toruloides* protein IDs of enzymes with predicted or demonstrated XR function.

Strains	Protein ID	Annotation	Expression level on xylose (versus glucose)	Source
*R. toruloides* IFO0880	RTO4_9774	Xylose reductase	2.6-fold increase (transcription)	Jagtap et al. (2021), Adamczyk et al. (2023)
*R. toruloides* IFO0880	RTO4_11553	Aldose reductase	1.2-fold increase (transcription)	Jagtap et al. (2019), Jagtap et al. (2021)
*R. toruloides* IFO0880	RTO4_11882	Glycerol 2-dehydrogenase	2.7-fold increase (transcription)	Protzko et al. (2019), Jagtap et al. (2021)
*R. toruloides* IFO0880	RTO4_13562	Alcohol dehydrogenase	1.4-fold increase (transcription)	Jagtap et al. (2021)
*R. toruloides* CBS14	Rhto_03963	Xylose reductase	36-fold increase (protein)	Tiukova et al. (2019)
*R. toruloides* CBS14	Rhto_07387	Xylose and arabinose reductase	36-fold increase (protein)	Tiukova et al. (2019)
*R. toruloides* CBS14	Rhto_00641	Aldo/keto reductase	34-fold increase (protein)	Tiukova et al. (2019)
*R. toruloides* CBS14	Rhto_06555	Aldo/keto reductase	32-fold increase (protein)	Tiukova et al. (2019)
*R. toruloides* CBS14	Rhto_00602	Aldo/keto reductase	31-fold increase (protein)	Tiukova et al. (2019)

XRs in *R. toruloides* might use NADPH as a cofactor, as several observations support this claim. RTO4_11553 (the key enzyme in galactose metabolism) showed activity with NADPH as a coenzyme but not with NADH (Jagtap et al. 2019). From the multi-omics study, all other potential XRs, including RTO4_9774 (annotated XR), RTO4_11882 (annotated as glycerol 2-dehydrogenase), and RTO4_13562 (annotated as alcohol dehydrogenase), were predicted to be NADPH dependent (Kim et al. 2020). RHTO_03963 (annotated as xylose reductase; NCBI accession number XP_016271038) uses NADPH as a cofactor during xylose cultivation based on genome-scale metabolic network simulation (Pinheiro et al. 2020). Yeasts have multiple mechanisms to regenerate NADPH in the cytosol, including metabolic reactions of malic enzyme and isocitrate dehydrogenase. The expression level of malic enzyme (*ME1*) and isocitrate lyases (*ICL1* and *ICL2*) was higher under xylose than glucose conditions, according to the transcriptomic data (Jagtap et al. 2021). This coincides with a genome-scale metabolic model, indicating malic enzyme as a major NADPH regeneration route (Rekena et al. 2023). Additionally, when *R. toruloides* was grown on xylose, increased mRNA levels of putative NADP⁺-dependent alcohol dehydrogenases have been observed. This upregulation may be a cellular response to balance cytosolic NADPH levels (Tiukova et al. 2019). Notably, NADPH is essential for lipogenesis, meaning that XR activity and natural lipid generation must compete for the same NADPH pool (Yu et al. 2018). Altering XR cofactor preference from NADPH to NADH was successfully explored in *Sa. cerevisiae* to improve xylose fermentation under oxygen-limited conditions (Watanabe et al. 2007, Ha et al. 2010). Therefore, a similar approach—introducing an XR variant favoring NADH—could be applied in *R. toruloides* to investigate its impact on NADPH balance within the cell and its impact on lipid production.

### Xylitol dehydrogenase

The next step of xylose metabolism is the oxidation of xylitol into xylulose by XDH (Toivari et al. 2004). *R hodotorula toruloides* grown on xylose accumulate increased levels of intracellular xylulose, proposing the existence of functioning XDH in *R. toruloides* (Jagtap et al. 2021). Multiple genes are potentially encoding for putative XDH in *R. toruloides* (Kim et al. 2020, Jagtap et al. 2021).

Among these putative XDHs in *R. toruloides*, RTO4_8988 (NCBI accession number PRQ77920) has been highlighted in several studies as an XDH. A multi-omics study predicted that RTO4_8988 plays a role in catabolizing various substrates, including xylose and xylulose (Kim et al. 2020). The transcription levels of RTO4_8988 increased nine-fold under xylose conditions compared to glucose conditions (Jagtap et al. 2021). Adamczyk et al. (2023) further confirmed the function of the RTO4_8988 gene in *R. toruloides* IFO0880 by deleting it, which resulted in significantly delayed growth on xylose and a marked increase in xylitol accumulation compared to the wild type, confirming the function of RTO4_8988 in xylose metabolism.

Two additional genes, RTO4_16452 (NCBI accession number PRQ72528) and RTO4_12977 (NCBI accession number PRQ75955), have also been proposed to encode XDH. RTO4_16452 (annotated as D-xylulose reductase from the study) is a homolog of NADH-dependent XDHs in other fungal species, such as *Aspergillus oryzae* and *Aspergillus niger* (Adamczyk et al. 2023). RTO4_12977 (annotated as L-iditol 2-dehydrogenase from the study) has similarity with xylitol dehydrogenase (*XYL2*) of *Sa. cerevisiae* and is a homolog of L-arabitol-4-dehydrogenase that shows putative XDH activities in *A spergillus oryzae* (Kim et al. 2020). Under xylose conditions, both genes show significant transcriptional upregulation compared to glucose conditions: a 42-fold increase for RTO4_16452 and a 55-fold increase for RTO4_12977 (Jagtap et al. 2021).

Deletion of either RTO4_16452 (annotated as D-xylulose reductase) or RTO4_12977 (annotated as L-iditol 2-dehydrogenase) did not affect the growth of xylose and xylitol. However, the RTO4_16452 deletion mutant was unable to grow on L-arabitol, suggesting that RTO4_16452 primarily functions as an L-arabitol-4-dehydrogenase while also exhibiting promiscuous XDH activity. Notably, when the deletion mutant of RTO4_16452 was grown on xylose, it accumulated more xylitol than the wild-type *R. toruloides*, supporting that the promiscuous activity of RTO4_16452 as an XDH (Adamczyk et al. 2023). Collectively, these findings suggest the existence of multiple enzymes showing XDH activities in *R. toruloides*. Enzymes proposed to exhibit XDH activity are summarized in Table 2.

**Table 2.** *R hodotorula toruloides* protein IDs of enzymes with predicted or demonstrated XDH function.

Strains	Protein ID	Annotation	Expression level on xylose (versus glucose)	Deletion mutant grown with xylose	Source
*R. toruloides* IFO0880	RTO4_8988	Xylitol dehydrogenase	9.2-fold increase (transcription)	No growth	Jagtap et al. (2021), Adamczyk et al. (2023), Kim et al. (2020)
*R. toruloides* IFO0880	RTO4_16452	D-xylulose reductase	1.6-fold increase (transcription)	No impact on growth	Jagtap et al. (2021), Adamczyk et al. (2023), Kim et al. (2020)
*R. toruloides* IFO0880	RTO4_12977	L-iditol 2-dehydrogenase	55-fold increase (transcription)	No impact on growth	Jagtap et al. (2021), Adamczyk et al. (2023), Kim et al. (2020)
*R. toruloides* CBS14	Rhto_01970	Xylitol dehydrogenase	36-fold increase (protein)	N/A	Tiukova et al. (2019)
*R. toruloides* CBS14	Rhto_01629	L-iditol 2-dehydrogenase	36-fold increase (protein)	N/A	Tiukova et al. (2019)

N/A: not available.

### Xylulokinase

XK is a pivotal enzyme for efficient xylose utilization in a xylose-fermenting yeast such as *Sc. stipitis* (Jin et al. 2002). XK phosphorylates xylulose into xylulose-5-phosphate, which enters the central carbon metabolism through the PPP (Richard et al. 2000). However, RTO4_16850 (NCBI accession number PRQ72926) annotated as XK in *R. toruloides* is not required for growth on xylose. Furthermore, RTO4_16850 was not expressed under glucose and xylose conditions (Jagtap et al. 2021). Further investigation revealed that no corresponding peptides of RTO4_16850 were detected in xylose-grown cells (Kim et al. 2020). This absence of XK protein has also been confirmed in *R. toruloides* CCT7815 strain (Rekena et al. 2023). These findings suggest that XK is not expressed under xylose conditions, implying that xylulose may enter central carbon metabolism through an alternative route that bypasses the canonical xylulose-5-phosphate step.

A more thorough investigation is needed to determine whether the native XK in *R. toruloides*, RTO4_16850, is functionally active. Another oleaginous yeast, *Y. lipolytica*, similarly showed negligible XK expression even in xylose-consuming cells (Ledesma-Amaro et al. 2016). While *Y. lipolytica* has a complete oxidoreductive xylose pathway consisting of XR (YALI0D07634), XDH (YALI0E12463), and XK (YALI0F10923), it cannot grow on xylose as the sole carbon source (Niehus et al. 2018). In quantitative polymerase chain reaction analyses, the native XK showed the lowest transcription level among xylose-metabolic enzymes (YALI0D07634 and YALI0E12463) and is not induced by xylose (Ledesma-Amaro et al. 2016). Nevertheless, the native XK (YALI0F10923) is functional. When a codon-optimized YALI0F10923 was expressed in a mutant of *Escherichia coli* BW25113 lacking a *XylB* (which encodes XK), it resorted to growth on xylose (Rodriguez et al. 2016).

Moreover, previous studies have reported the successful construction of a xylose utilization pathway in *Y. lipolytica* by overexpressing the native XK, along with its endogenous or *Sc. stipitis* XR and XDH (Ledesma-Amaro et al. 2016, Rodriguez et al. 2016, Wu et al. 2019, Prabhu et al. 2020, Yook et al. 2020). If the endogenous *R. toruloides* XK (RTO4_16850) is indeed a functional enzyme, increasing its expression level will channel the xylulose flux into xylulose-5-phosphate, achieving a more streamlined xylose pathway.

### D-arabitol from xylose

*Rhodotorula toruloides* produces D-arabitol as a byproduct of xylose metabolism. While D-arabitol can be considered a valuable chemical, its accumulation hinders efficient xylose utilization. Therefore, reducing D-arabitol accumulation is essential for improving xylose uptake and maximizing yields of target products. A deeper understanding of why and how *R. toruloides* produces D-arabitol is key to optimizing xylose metabolism.

Most xylose-fermenting yeasts produce xylitol as a byproduct of xylose metabolism, which is due to intracellular redox imbalance. While XR reduces xylose into xylitol using NADPH as a cofactor, XDH oxidizes xylitol to xylulose using NAD^+^ as a cofactor. This cofactor difference leads to the accumulation of NADH in the cytosol, requiring NAD^+^ regeneration for redox balance (Ostergaard et al. 2000). Since XDH catalyzes a reversible reaction, yeast can turn xylulose back into xylitol when excess NADH is accumulated to restore NAD^+^ levels (Wahlbom and Hahn-Hagerdal 2002). This is especially prominent under oxygen-limited conditions, where NADH oxidation via respiration is restricted (Zhang et al. 2012).

*Rhodotorula toruloides* has a distinct trait of D-arabitol accumulation from xylose (Jagtap and Rao 2018, Pinheiro et al. 2020, Rekena et al. 2023). Stereochemical analyses confirmed that the arabitol produced is D-form, suggesting that it is produced from xylose (Adamczyk et al. 2023, Rekena et al. 2023). *R hodotorula toruloides* reduces xylulose to D-arabitol by D-arabitol 4-dehydrogenase (DA4DH). Other xylose-fermenting yeasts also have the potential to reroute xylulose to D-arabitol. For example, *Sc. stipitis* possesses a D-arabitol-specific polyol dehydrogenase (Hallborn et al. 1995). However, a wild-type *Sc. stipitis* does not accumulate D-arabitol because it has a functional XK, whereas the XK deletion mutant accumulates both xylitol and D-arabitol. Nonetheless, the predominant accumulated polyol by the XK-deleted mutant was xylitol (Jin et al. 2005). This suggests that *Sc. stipitis* does not prefer D-arabitol production to xylitol production regardless of XK activity (Jin et al. 2002). Intriguingly, *R. toruloides* accumulates D-arabitol much more than xylitol.

Since xylulose is a substrate for both XDH and DA4DH, it is speculated that the kinetic properties of the enzymes in *R. toruloides* might explain these phenotypic differences: xylitol versus D-arabitol production. If DA4DH exhibits a higher substrate affinity for xylulose than XDH, *R. toruloides* could accumulate D-arabitol rather than xylitol. *Rhodotorula toruloides* has multiple putative XDHs. However, deletion of these putative XDHs did not impair *R. toruloides* growth on xylose (Adamczyk et al. 2023). Although enzyme properties of endogenous XDHs have not been characterized, these enzymes might exhibit low substrate affinities for xylulose. Further studies on the enzyme properties of XDHs and DA4DH in *R. toruloides* are necessary to elucidate the metabolic mechanism driving D-arabitol accumulation.

Overexpressing a transcription factor showed a reduction in D-arabitol formation. When RTO4_12978 (annotated as fungal specific transcription factor domain-containing protein; NCBI accession number PRQ75956), named Pnt1, was overexpressed, the mutant accumulated less D-arabitol from 40 g l^−1^ xylose. Further investigation showed that overexpression of RTO4_12978 increased protein levels related to PPP and xylose metabolisms, such as RTO4_9774 (annotated as XR) and RTO4_8988 (annotated as XDH) (Coradetti et al. 2023). Investigating cellular mechanisms beyond each responsible enzyme could be the key to understanding D-arabitol accumulation.

### D-arabitol 4-dehydrogenase

In the *R. toruloides* IFO0880 strain, RTO4_8988 (annotated as XDH) was proposed to function as a DA4DH. Although RTO4_8988 was highlighted to function as an XDH in the previous section, the phenotype after deletion suggests its DA4DH function. A deletion mutant of RTO4_8988 showed poor growth on xylose and accumulated xylulose, supporting that RTO4_8988 functions as DA4DH. In the deletion mutant, rerouting accumulated xylulose via a codon-optimized *Aspergillus niger* XK restored growth on xylose. This recovery further supports that RTO4_8988 is primarily responsible for reducing xylulose to D-arabitol. Notably, the deletion mutant showed elevated xylitol accumulation, suggesting a promiscuous activity of RTO4_8988 as XDH. However, the mutant was able to reassimilate xylitol, foreshadowing the existence of another enzyme working as XDH (Adamczyk et al. 2023). D-arabitol-specific polyol dehydrogenase from *Sc. stipitis* is also reported to have an XDH activity (Hallborn et al. 1995). RTO4_8988 is most likely a DA4DH, yet it has activity as an XDH in *R. toruloides*.

More than one enzyme may facilitate the conversion of xylulose into D-arabitol. In the *R. toruloides* CCT7815 strain, two putative DA4DH encoding genes, RHTO_07844 (NCBI accession number XP_016274093) and RHTO_07702 (NCBI accession number XP_016273951), have been annotated as D-arabitol dehydrogenase. However, only RHTO_07844 was detected under xylose conditions, indicating its essential role in xylose metabolism (Rekena et al. 2023). Genome sequence analyses suggest that RTO4_8905 (annotated as short-chain dehydrogenase; NCBI accession number PRQ77837) has a high level of identity with DA4DH in *A spergillus oryzae* (Kim et al. 2020). It coincides with a highly elevated transcription level (21-fold) under xylose conditions (Jagtap et al. 2021). Another potential DA4DH, RTO4_9837 (annotated as hypothetical protein; NCBI accession number PRQ71722), was identified by a BLAST search of *Aspergillus niger* DA4DH (An04g09410) against the *R. toruloides* genome (Kim et al. 2020). The transcription level of RTO4_9837 was slightly increased (1.5-fold) under xylose conditions (Jagtap et al. 2021). No prior study has examined the impacts of DA4DH overexpression on xylose utilization in *R. toruloides*, but it is worthwhile to investigate. All putative DA4DHs identified in *R. toruloides* are listed in Table 3.

**Table 3.** *Rhodotorula toruloides* protein IDs of enzymes with predicted or demonstrated DA4DH function.

Strains	Protein ID	Annotation	Expression level on xylose (versus glucose)	Deletion mutant grown with xylose	Source
*R. toruloides* IFO0880	RTO4_8988	Xylitol dehydrogenase	9.2-fold increase (transcription)	No growth	Jagtap et al. (2021), Adamczyk et al. (2023), Kim et al. (2020)
*R. toruloides* IFO0880	RTO4_8905	D-Arabitol 4-Dehydrogenase	21-fold increase (transcription)	N/A	Jagtap et al. (2021), Kim et al. (2020)
*R. toruloides* IFO0880	RTO4_9837	D-Arabitol 4-Dehydrogenase	1.5-fold increase (transcription)	N/A	Jagtap et al. (2021), Kim et al. (2020)
*R. toruloides* CCT7815	Rhto_01 970	D-arabitol dehydrogenases		N/A	Rekena et al. (2023)

N/A: not available.

### D-arabitol 2-dehydrogenase

D-arabitol accumulation and its re-assimilation upon xylose depletion are hallmarks of xylose metabolism in *R. toruloides*. To re-assimilate D-arabitol, it needs to be converted into D-ribulose, which can then be phosphorylated into D-ribulose 5-phosphate, entering PPP. This conversion of D-arabitol into D-ribulose, catalyzed by D-arabitol 2-dehydrogenase (DA2DH), has been reported in *Candida albicans* (Wong et al. 1993). Additionally, L-xylulose reductase has been suggested to convert D-arabitol into D-ribulose in *Ambrosiozyma monospora* (Verho et al. 2004).

RTO4_9990 in *R. toruloides* IFO0880 is a strong candidate for DA2DH. Under xylose conditions, the transcription level of RTO4_9990 (annotated as hypothetical protein; NCBI accession number PRQ71875) increased 27-fold (Jagtap et al. 2021). Deletion of RTO4_9990 resulted in growth defects on D-arabitol, whereas both the mutant and the wild type showed comparable growth on D-ribulose. This finding supports the role of RTO4_9990 in converting D-arabitol into D-ribulose, facilitating D-arabitol utilization via PPP in *R. toruloides* (Adamczyk et al. 2023).

Enzymes annotated as L-xylulose reductase may function as DA2DH. In *R. toruloides* CCT7815, the protein level of RHTO_00373 (NCBI accession number XP_016277064), annotated as an L-xylulose reductase, increased 10-fold under xylose conditions, suggesting a potential role as a DA2DH (Rekena et al. 2023). Similarly, in *R. toruloides* IFO0880, RTO4_16452, also annotated as L-xylulose reductase from this study, showed a 42-fold increase in transcription levels under xylose conditions as compared to glucose conditions (Jagtap et al. 2021). However, deletion of RTO4_16452 had no impact on growth with D-arabitol as the sole carbon source (Adamczyk et al. 2023). As previously described, RTO4_16452 primarily functions as an XDH and is unlikely to act as a DA2DH in *R. toruloides*. Further investigation is warranted to identify and characterize DA2DH in *R. toruloides*.

### Ribulokinase

D-ribulose is phosphorylated into ribulose 5-phosphate by RK, linking the non-oxidative PPP with central carbon metabolism. RK has been identified in the model yeast, *S a. cerevisiae*. Ydr109c, a protein with a previously unknown function, was confirmed to function as RK. Deletion of YDR109C led to D-ribulose accumulation from glucose, as confirmed by a ^13^C-labeled glucose experiment (Singh et al. 2017).

In *R. toruloides* IFO0880, RTO4_14368 (annotated as putative sugar kinase; NCBI accession number PRQ75346) is the ortholog of RK in *Sa. cerevisiae* (Adamczyk et al. 2023). The deletion mutant of RTO4_14368 failed to grow on D-ribulose, confirming its role as RK (Adamczyk et al. 2023). Additionally, the transcription level of RK increased 10-fold under xylose conditions compared to glucose conditions (Jagtap et al. 2021). The deletion mutant of RTO4_14368 was not able to grow on D-arabitol and xylose, suggesting that RK is essential for xylose metabolism in *R. toruloides*. Because D-arabitol is an intermediate of xylose catabolism, the growth defect of the deletion mutant suggests there is no redundant enzyme capable of substituting for RK activity (Adamczyk et al. 2023). As XK activity is a pivotal enzyme in xylose metabolism in *Sc. stipitis*, RK might control xylose utilization in *R. toruloides*. Therefore, overexpression of RK is a potential target for improving xylose utilization in *R. toruloides*.

### Bioconversion of cellulosic hydrolysates by *R. toruloides*

Many *R. toruloides* strains (IFO0880, CBS14, CCT0783, CCT7815, and 1588) have been isolated and evaluated for their natural ability to convert glucose, xylose, and mixed sugar or hydrolysate substrates into products such as lipids and carotenoids, without the need for metabolic engineering (Jagtap and Rao 2018, Tiukova et al. 2019, Pinheiro et al. 2020, Monteiro de Oliveira et al. 2021, Osorio-González et al. 2023). With metabolic engineering, *R. toruloides* has been further optimized to produce a wide range of acetyl–CoA-derived products, including fatty alcohols, polyketides, terpenes, and organic acids, from mixed glucose and xylose or lignocellulosic hydrolysates (Geiselman et al. 2020, Cao et al. 2022, Lin et al. 2022, Otoupal et al. 2022, Liu et al. 2023, Adamczyk et al. 2025). A summary of these examples is provided in Table 4.

**Table 4.** Sugar consumption rate by various *R. toruloides* strains cultured in mixed sugar or cellulosic hydrolysate media.

Strains	Substrates	Sugar uptake rate (g l^−1^ h^−1^)	D-arabitol formation	Product titer	Reference
*R. toruloides* IFO0880	Yeast extract, peptone 150 g l^−1^ xylose	Xylose: 0.42	33 g l^−1^	DCW: 23 g l^−1^ Lipid: 4.4 g l^−1^	Jagtap and Rao (2018)
*R. toruloides* IFO0880-ADS	Sorghum hydrolysate Total sugar: 80 g l^−1^	Glucose: 0.41 Xylose: 0.11	ND	Lipid: 8.5 g l^−1^	Woodruff et al. (2023)
*R. toruloides* IFO0880-ADS	Sorghum hydrolysate Starting sugar: 40 g l^−1^	Glucose: 0.38 Xylose: 0.22	ND	Lipid: 16.7 g l^−1^	Woodruff et al. (2023)
*R. toruloides* CBS14	Xylose 40 g l^−1^ C:N ratio: 75	Xylose: 0.29	ND	DCW: 11.1 g l^−1^ Lipid: 4.2 g l^−1^	Tiukova et al. (2019)
*R. toruloides* CCT 0783	Xylose: 70 g l^−1^	Xylose: 0.73	23 g l^−1^	Lipid: 8.1 g l^−1^	Pinheiro et al. (2020)
*R. toruloides* CCT 7815	C5-birch hydrolysate Xylose: 48 g l^−1^ Glucose: 14 g l^−1^ Others: 19 g l^−1^	Xylose: 1.20 Glucose: 0.96 Others: 0.50	8.4 g l^−1^	Lipid: 11 g l^−1^ Carotenoid: 1.7 mg l^−1^	Monteiro de Oliveira et al. (2021)
*R. toruloides* 1588	Xylose: 25 g l^−1^ Glucose: 25 g l^−1^ C:N ratio: 70	Total sugar: 0.33	ND	DCW: 13.33 g l^−1^ Lipid: 5.18 g l^−1^	Osorio-González et al. (2023)
*R. toruloides* IFO0880 maquFOH	Poplar hydrolysate	ND	ND	Fatty Alcohol: 87 mg l^−1^	Lin et al. (2022)
*R. toruloides* IFO0880 I12	Yeast extract, peptonexylose: 6 g l^−1^ Glucose: 14 g l^−1^	ND	ND	Triacetic acid lactone: 2.2 g l^−1^	Cao et al. (2022)
*R. toruloides* IFO0880 *2-PSG*	Sorghum hydrolysate Xylose: 20 g l^−1^ Glucose: 38 g l^−1^	ND	ND	Triacetic acid lactone: 3.9 g l^−1^	Otoupal et al. (2022)
*R. toruloides* IFO0880 MCR-ALD6-g2945	* DMR hydrolysate Xylose : 34 g l^−1^ Glucose: 65 g l^−1^	ND	ND	3-hydroxypropinoic acid: 19 g l^−1^	Liu et al. (2023)
*R. toruloides* IFO0880 ABFPUB_26	* DMR hydrolysate Xylose : 37 g l^−1^ Glucose: 76 g l^−1^	Xylose: 0.47 Glucose: 0.18	ND	*ent*-kaurene: 1.4 g l^−1^	Geiselman et al. (2020)
*R. toruloides* IFO0880 B22	* DMR hydrolysate Total sugar: 194 g	ND	ND	(E)-α-bisabolene: 18 g l^−1^	Adamczyk et al. (2025)

* DMR hydrolysate: lignocellulosic hydrolysate from corn stover, prepared by deacetylation, mechanical refining, and enzymatic hydrolysis.

ND: no data.

Lipid production using hydrolysates containing xylose has been reported. From sorghum hydrolysate, *R. toruloides* IFO0880 overexpressing acetyl–CoA carboxylase, diacylglycerol acyltransferase (DGA1), and stearoyl-CoA desaturase (SCD) yielded lipid up to 0.11 g g^−1^ sugar, while fed-batch fermentation yielded 0.16 g g^−1^ sugar. However, the specific growth rate was lower in xylose (0.013 h^−1^) compared to glucose (0.052 h^−1^) (Woodruff et al. 2023). By optimizing the growth media with a glucose-to-xylose ratio of 1:1, a C:N ratio of 70, and the addition of dibasic sodium phosphate as an inducer, *R. toruloides* 1588 achieved a lipid yield of 0.28 g g^−1^ sugar from wood hydrolysates (Osorio-González et al. 2023). Diáz et al. (2018) tested promoter strength and found that *XYL1* has the highest expression level on xylose in *R. toruloides* CECT13085. By overexpressing *DGA1* and *SCD* with *XYL1* promoter, combined with an adaptive laboratory evolution, they achieved the lipid yield of 0.18 g g^−1^ sugar from a lignocellulosic hydrolysate without detoxification. *R hodotorula toruloides* CCT7815 produced carotenoids (1.7 mg l^−1^) on birch hydrolysate (Monteiro de Oliveira et al. 2021). These findings underscore the versatility and robustness of *R. toruloides* strains in bioconversion processes, particularly for lignocellulosic substrates and mixed sugar sources.

Many *R. toruloides* strains have been engineered to produce modified lipids and acetyl–CoA-derived products from xylose or xylose-containing hydrolysates. From engineered poplar hydrolysates, ~87 mg l^−1^ of fatty alcohol was produced by engineered *R. toruloides* strain (Lin et al. 2022). Cao et al. (2022) demonstrated the conversion of 14 g l^−1^ glucose and 6 g l^−1^ xylose into 2.2 g l^−1^ triacetic acid lactone (TAL), an acetyl–CoA-derived polyketide. Using unfiltered one-pot pretreatment and saccharification of sorghum biomass, an engineered *R. toruloides* produced 3.9 g l^−1^ of TAL, showcasing the industrial potential of this process. This medium contained 38 g l^−1^ glucose and 20 g l^−1^ xylose, with high levels of acetate (15 g l^−1^) and lactate (15 g l^−1^) (Otoupal et al. 2022). An engineered *R. toruloides* strain produced 1.4 g l^−1^  *ent*-kauren from corn stover hydrolysate, prepared by deacetylation, mechanical refining, and enzymatic hydrolysis (DMR hydrolysate) (Geiselman et al. 2020). Adamczyk et al. (2025) achieved 18 g l^−1^ of (E)-α-bisabolene production from DMR hydrolysates, with pure glucose feeding, from 194 g of total sugar utilized. Additionally, the strain harbors 3-hydroxypropionic acid (3-HP) producing pathway from acetyl–CoA that produced 19 g l^−1^ of 3-HP from DMR hydrolysate. The culture media contained 65 g l^−1^ glucose and 34 g l^−1^ xylose (Liu et al. 2023). These findings highlight the bioconversion potential of lignocellulosic hydrolysates into value-added chemicals by *R. toruloides* strains.

However, xylose metabolism in *R. toruloides* is inefficient, as they still produce D-arabitol up to 33 g l^−1^ as a byproduct (Table 4). Lipid accumulation (up to 11 g l^−1^) is often linked to a high carbon-to-nitrogen ratio (C:N ratio). When a complex medium with yeast extract and peptone is used with 150 g l^−1^ of xylose, a high dry cell weight (23 g l^−1^) is observed (Jagtap and Rao 2018). Metabolic engineering efforts to increase xylose uptake and reduce D-arabitol formation are crucial for improving the bioconversion of cellulosic hydrolysates.

## Conclusion

In this review, we discussed how various enzymes in *R. toruloides* are used to catabolize xylose, an abundant sugar derived from plant biomass. Multiple opportunities exist to improve the efficiency of xylose utilization by engineering *R. toruloides* capable of simultaneously consuming multiple sugars. Xylose may represent a preferred sugar for producing novel bioproducts from renewable biomass, as it is often easier to redirect metabolic flux away from ethanol and toward other desired products during growth on xylose than on glucose. To fully harness this potential, key metabolic engineering targets include minimizing D-arabitol accumulation and enabling the co-consumption of glucose, xylose, and acetate. Achieving these is critical for the complete and efficient use of lignocellulosic sugars, harnessing the full potential of this promising yeast.

There is accumulating evidence that *R. toruloides* has a non-conventional xylose metabolism. While most yeast utilize XK to convert xylulose into xylulose-5-phosphate, *R. toruloides* does not express a gene coding for XK under xylose conditions. Instead, xylulose is converted into D-arabitol. There are multiple reports of D-arabitol accumulation from xylose by *R. toruloides*. The presence of this alternative xylose pathway has been confirmed at the transcriptional, proteomic, and phenotypic levels.

Xylose catabolism in *R. toruloides* is mediated by enzymes with multi-functional roles at various metabolic steps, making enzyme annotation challenging. Moreover, multiple *R. toruloides* genome sequences and models, each with different protein ID naming conventions and annotations, have led to inconsistencies and confusion within the community. To address this, a more systematic and unified naming scheme for xylose-related genes is necessary, as it will help clarify enzyme functions and development of *R. toruloides* as robust microbial chassis for bioproduction.
